# Vaccine nationalism and internationalism: perspectives of COVID-19 vaccine trial participants in the United Kingdom

**DOI:** 10.1136/bmjgh-2021-006305

**Published:** 2021-10-18

**Authors:** Samantha Vanderslott, Katherine Emary, Rebecca te Water Naude, Marcus English, Tonia Thomas, Maia Patrick-Smith, John Henry, Naomi Douglas, Maria Moore, Arabella Stuart, Susanne H Hodgson, Andrew J Pollard

**Affiliations:** 1 Oxford Vaccine Group, Department of Paediatrics, University of Oxford, Oxford, Oxfordshire, UK; 2 NIHR Oxford Biomedical Research Centre, Oxford, Oxfordshire, UK; 3 Medical School, Medical Sciences Division, University of Oxford, Oxford, Oxfordshire, UK; 4 Jenner Institute, Nuffield Department of Medicine, University of Oxford, Oxford, Oxfordshire, UK

**Keywords:** COVID-19, health policy, public health, vaccines, qualitative study

## Abstract

**Background:**

Vaccine nationalism has become a key topic of discussion during the development, testing, and rollout of COVID-19 vaccines. Media attention has highlighted the ways that global, coordinated access to vaccines has been limited during the pandemic. It has also exposed how some countries have secured vaccine supply, through bilateral purchase agreements and the way pharmaceutical companies have priced, negotiated, and delivered these supplies. Much of the focus of this debate has been on the vaccine supply ‘winners’ and ‘losers’, but the voices of public opinion have been more limited.

**Methods:**

We explore the concepts of vaccine nationalism and internationalism from the perspective of vaccine trial participants, using an empirical perspectives study that involved interviews with phase I/II COVID-19 vaccine trial participants in Oxford, UK. We surveyed and interviewed participants between September and October 2020 about their views, motivations and experiences in taking part in the trial.

**Results:**

First, we show how trial participants describe national and international ideas about vaccination as intertwined and challenge claims that these positions are mutually exclusive or oppositional. Second, we analyse these viewpoints further to show that vaccine nationalism is closely connected with national pride and metaphors of a country’s scientific achievements. Participants held a global outlook and were highly supportive of the prioritisation of vaccines by global need, but many were also pessimistic that such a solution could be possible.

**Conclusion:**

Trial participants constitute an informed public group, with situated public expertise that the global community could draw on as an expert opinion. We argue that vaccine nationalism is strongly attached to national character and, therefore, it is more difficult for ownership of a vaccine to be thought of as international.

**Key questions**

**What is already known?**

- Vaccine nationalism is allowing higher income countries to secure supply of and access to COVID-19 vaccines to the detriment of lower income countries.
- Mechanisms for vaccine internationalism to achieve more equitable vaccine distribution (such as COVAX), face significant challenges.
- National surveys describe public views about COVID-19 vaccine priorities and access, but more detailed qualitative interviews have not yet been pursued.

**What are the new findings?**

- Surveys and interviews with COVID-19 vaccine trial participants reveal a national pride in vaccines developed in the UK, however, many also support the global supply of vaccines according to need.
- While participants have high global aspirations for access to vaccines, they also note the difficulty of achieving such ideals in reality.

**What do the new findings imply?**

- Vaccine trial participants are an informed public group which could be drawn on as a situated public expertise.
- Vaccine nationalism, through a national character attached to vaccines, makes it harder to attain an internationalownership of vaccines.

## Introduction

### COVID-19 vaccine nationalism

The COVID-19 pandemic has significantly impacted the entire world. Many recognise that the control of the pandemic relies on the management of the disease in all countries: ‘The world won’t be safe while any single country is still fighting the virus’.^1^ Vaccines are widely thought to be at the forefront of a solution to the global pandemic. Governments around the world must try to provide enough vaccines for their populations at a time when the consequences of their actions and inactions have rarely been so swiftly and starkly demonstrated than in case and mortality figures.

‘Vaccine nationalism’ most commonly refers to the pursuit of vaccines in the national interest, for example, through supply agreements or export bans, including where this might be to the detriment of other countries. Bollyky and Brown writing in *Foreign Affairs* call it a ‘my country first’ approach.^2^ The reasons for such a response to vaccine procurement and rollout have been framed through the lens of national security through a ‘medicalisation of insecurity’.^3^ An alternative response would be one in the pursuit of global health that employs more coordinated and unified ways of dealing with disease. For vaccines during an epidemic, the conditions of limited or slow supply mean that a security imperative of being able to procure vaccines as a priority and/or in larger quantities for some countries will negatively impact others. Indeed, some high-income countries (HICs) have purchased sufficient vaccines for their populations several times over.^4^ In the early stages of development, this was a rational approach, since not all vaccines will reach the market but since vaccines have been approved and supply is finite, most doses have, thus, far been given in HICs, inevitably leading to inequitable distribution of vaccines.^4^ Now HICs are rolling out campaigns of third-dose boosters and vaccinating children.

Low-income countries (LICs) are often constrained in being able to negotiate favourable vaccine supply arrangements and additionally may not have the infrastructure to develop local manufacturing capability. The WHO has warned to guard against vaccine nationalism or face further virus transmission with the inevitable emergence of new variants and urges HICs to share vaccines globally once priority health workers and at-risk groups are vaccinated – through initiatives like COVAX.^4^ COVAX is a partnership between the World Health Organization (WHO), Gavi, the Vaccine Alliance and the Coalition for Epidemic Preparedness Innovations, which was established as ‘a global risk-sharing mechanism for pooled procurement and equitable distribution of COVID-19 vaccines’.^5^

Technology transfer and open intellectual property (IP) access could also improve access to vaccines. As part of COVAX, the COVID-19 Technology Access Pool (C-TAP) exists to share knowledge, intellectual property and data related to COVID-19 health technologies, however, this initiative has not gained significant traction with COVID-19 vaccine manufacturers, who have not engaged with C-TAP.^6^ Of course, part of the opposition is the threat to profits. However, it should also be noted that there are some risks in open sharing of IP and manufacturing know-how without adequate controls, as manufacture of inferior products could damage vaccine confidence. Similarly, countries have shown support waiving a World Trade Organization (WTO) rule that protects the IP of COVID-19 vaccines and treatments.^6^

Concerns, as voiced by Hassoun^7^, are that LICs will lack access to vaccination, and COVAX will be unable to ensure global distribution. For example, countries have arranged bilateral agreements with pharmaceutical companies even if they have joined COVAX. It means that only 20% of low and middle-income country (LMIC) populations stand to benefit from COVAX-provided COVID-19 vaccines.^8^ Even across HICs and some MICs, competition in negotiations and what is considered fair conduct has also presented a challenge. The US began early with vaccine nationalism rhetoric and policy through the Trump administration. The export of vaccines and raw materials essential to vaccine production, such as bags and filters, was restricted through a Defense Production Act (DPA), which compels companies to fulfil federal orders ahead of commercial orders, resulting in worldwide shortages.^9^ While in Europe, a disagreement between AstraZeneca and the European Union (EU) began over vaccine supplies, which culminated with the EU threatening to permit member states to restrict the supply of vaccines produced in their countries to the UK.^10^ The first EU country to block the export of vaccines was Italy, which blocked a shipment of Oxford-AstraZeneca vaccine in March 2021.^11^ It is clear that these political moves arise because of supply concerns, highlighting the somewhat predictable challenge of upscaling manufacturing of any biological product to the scale required to vaccinate a high proportion of adults on the planet. Indeed, while very uneven, it is still extraordinary that over 6 billion does of SARS-CoV-2 vaccine have been delivered as 2021 draws to an end.^12^

An alternative to vaccine nationalism is 'vaccine internationalism', as exemplified by the aims of COVAX. Vaccine internationalism suggests that vaccines should be available according to need at the international level through multilateral cooperation.^13 14^ The Association of Schools for Public Health in the European Region released a statement on vaccine internationalism, highlighting that coordination as well as equitable access is important and that ‘chaotic differences in vaccination policies both between and within countries… threaten our collective ability to control and suppress the virus worldwide’.^15^

The two related concepts of ‘vaccine diplomacy’ and ‘medical internationalism’ integrate health policy with foreign policy. Vaccine diplomacy is a mediating concept between nationalism and internationalism, where national strategic interests are pursued alongside an outward focus on donating and selling vaccines to other countries.^16^ Medical internationalism is a closely related concept, with the common example of Cuban doctors being posted abroad to provide support during health crises, including the COVID-19 pandemic. Since 1960, it is estimated that one in every five Cuban doctors have worked overseas for some time.^17^ Donation of vaccines has also historically taken place, such as the donation of millions of doses of a meningococcal vaccine (during the 1990s, Cuba was the first country to develop and produce a meningococcal A vaccine for meningitis following an outbreak in Uruguay).^18^ This activity stems from a ‘policy of proletarian internationalism and solidarity with other countries seeking to emerge from underdevelopment’.^17^

Even though these activities are outwardly facing, they can be argued to have national interests at heart. China, India and Russia have all been engaging in what has most popularly been termed ‘vaccine diplomacy’, where countries seek to expand their influence, either through grants, commercial contracts or COVAX. China has supplied vaccines to countries in Africa, Southeast Asia and Latin America; Russia to Eastern Europe and Central Asia; India to neighbouring countries, with the exception of Pakistan.^19^ The Serum Institute of India (SII) is the world’s largest vaccine maker by volume, manufacturing vaccines on license for pharmaceutical companies AstraZeneca and Novavax. India is also manufacturing their own vaccine through Bharat Biotech. Through COVAX, SII and the Oxford-AstraZeneca vaccine are the largest contributors of vaccines to LMICs thus far.^4^ However, India saw a domestic rise in COVID-19 cases during their second wave, leading to vaccine exports being halted temporarily.

Intentions aside, the ethically correct position on vaccine nationalism and internationalism has been debated. Ferguson and Caplan^20^ have defended vaccine nationalism based on the assertion that the first obligation of a country is to its citizens. Hassoun^7^ has responded to these claims with the argument that ‘luck of birth’ should not determine access to vaccines, which should be a basic human right. As she puts it: ‘vaccine nationalism is neither ethically justified, nor even in rich countries’ long-term self-interest’.^7^ The idea of moral duty as global citizens to look to the greatest need, regardless of distance or belonging to a national community, draws on a utilitarian philanthropic or public health ethic applied globally.^21^ Furthermore, as the UN Secretary-General António Guterres has repeatedly asserted, self-interests are also involved: ‘none of us is safe until we all are’.^22^

Citizens within a country cannot be protected completely against a threat, such as a virus, that does not respect borders. Taking a global public health perspective to solve the COVID-19 pandemic requires developing solutions that are not constrained by borders. A related but separate issue is that many HICs have now vaccinated significant proportions of their populations while some countries are yet to give a single dose. Commentators have argued that vaccines should be made widely available globally once vulnerable populations in HICs are protected.^1 23 24^ Public opinion is also mostly aligned.

The most comprehensive study on public opinion about the global rollout of COVID-19 vaccines^25^ surveyed 8209 participants from Australia, Canada, France, Italy, Spain, UK and US. They found that most agreed with the principle that allocation be based on need and were in favour of donation, while allocation based on the country in which vaccines were developed was least popular. The authors argued that global redistribution of vaccines has public support and that the economic and health costs of vaccine nationalism, including the impact on the global economy and the increased chance of novel variants, are of concern. Recently, a YouGov poll, conducted in the UK, asked whether Britain should prioritise the rollout of the vaccination in the UK, while India was experiencing high COVID-19 cases and deaths.^26^ The largest percentage (39%) thought the UK should be prioritised, even if this meant India was unable to obtain vaccine doses. However, the second largest group, 34% of those surveyed thought the UK should provide vaccine doses to India, even if it slowed down the rate of UK vaccination. This survey suggests that when confronted with a very realistic scenario, people may in fact be more likely to opt for a nationally-orientated view.

### Nationalist vaccines: the national character of vaccines

Before the COVID-19 pandemic, vaccine nationalism was already observed with other diseases. For example, HICs secured vaccines and pharmaceuticals for national use before international access, with ‘vaccines for smallpox and polio and drugs for HIV/AIDS’.^27^ In 2009, the novel H1N1 influenza virus or ‘swine influenza’ pandemic was a warning as to how countries might react to future pandemics. H1N1 influenza vaccines were preordered by HICs even before the WHO had declared a pandemic.^27^ Only when H1N1 influenza proved not to be as devastating as predicted did some HICs offer part of their stockpile to LICs. This experience may have informed the decision of global health agencies to establish COVAX in an attempt to encourage global equitable and coordinated access to vaccines.

Vaccine nationalism is most often associated with national control and ownership of vaccines. However, there is a secondary symbolic meaning by attaching a national ‘character’ to vaccines, often expressed through nationalist metaphors of winning and achievement. Vaccination against polio is one example: countries wanted to produce ‘their own’ polio vaccines rather than import from abroad. Even though the US was the first to develop a polio vaccine in 1955, the British health authorities, cautious after a high-profile US laboratory accident, created their version of inactivated polio vaccine a year later.^28^ This national attachment to vaccines has been reflected in polling about which COVID-19 vaccines Europeans trust the most, conducted in the context of vaccine supply concerns and the political backdrop of the UK’s exit from the EU. A YouGov Poll from March 2021 asked the question: ‘How safe, or unsafe, do you think the Pfizer-BioNtech/Oxford-AstraZeneca/Moderna vaccine is?’.^29^ UK trust in the safety of the Oxford-AstraZeneca vaccine developed by UK scientists was high with 77% of respondents viewing it as safe. In contrast in France, 23% of respondents who viewed it as safe, compared with 61% of respondents viewed it as unsafe. Even the Medicines and Healthcare products Regulatory Agency’s decision to restrict the vaccine to over-30-year olds had a minimal impact on safety perceptions, only dropping by 2% to 75% for UK respondents who considered the vaccine to be very or somewhat safe on 7–8 April 2021.^30^

National differences in public opinion are influenced by the countries that develop vaccines as well as backing by governments, public debate, regulatory authority responses and media coverage. As Van der Geest & Whyte^31^ describe, vaccines are similar to other pharmaceuticals in that they are also instruments of power. Vaccines not only have medical value but are metaphors that ‘facilitate particular social and symbolic processes’.^31^ They embody sociocultural ideas and relations via their promotion by governments. Vaccines as nationalist metaphors have been apparent through the older term of ‘medical nationalism’. Medical historians McMillen and Brimnes described medical nationalism through Indian opposition to a new tuberculosis vaccine, based on the fear of Indians being used as ‘guinea pigs’; in medical experimentation for the profit of governments, pharma companies and the international community.^32^ Medical nationalism was a reaction to the ‘application of outside knowledge via outside experts to solve an indigenous problem’,^32^ with vaccines representing interference from other countries. Vaccines are not free from context–they are closely associated with national ideals and interests. Promotion and resistance to vaccination programmes adds to the complexity.

Therefore, public opinion regarding vaccines matters. Through this paper, we look to those who were part of the vaccine development and made a contribution by actively volunteering to participate in a novel COVID-19 vaccine trial. We ascertained their views on national and international dimensions of COVID-19 vaccines, and where obligation lies for access and prioritisation of vaccines during a global pandemic. This perspective is novel, as we are not aware of a similar in-depth study of individuals about this topic, in realtime during a pandemic. We go further than the political, power-rooted definition of vaccine nationalism to explore the concept in a socio-cultural context that also wields symbolic power.

## Methods

This study is part of a large empirical study ‘COVQUAL’, which involved the surveying and semi-structured interviews with participants in COV001, a phase I/II vaccine trial in Oxford, UK of the novel COVID-19 vaccine ChAdOx1 nCoV-19 (NCT04324606).^33^ This ‘first-in-human,’ ‘multisite’ clinical trial is ongoing, having started in April 2020. The objective of COVQUAL is to understand the motivations, experiences and views of participants. We recruited from a purposeful sample of 770 healthy volunteers aged 18–55 years who were enrolled in COV001 and gave consent to be contacted about further research. This was a purposeful sample as we only contacted those who had enrolled in the phase I/II vaccine trial in Oxford and who also agreed to be contacted. We included no other additional criteria—for example, for demographic constitution of the sample.

A survey was sent in September 2020, and interviews were conducted between 18 September and 30 October 2020 by a team of 11 researchers (one social scientist, one public engagement manager, three clinicians, two research nurses and four medical students). Interviews took between 45 mins and 1.5 hours to complete. During the survey and interview period, COVID-19 cases in the UK had fallen after the first wave but were beginning to rise again. Also, at the time of interviewing, results of the COV001 trial had been released showing that the ChAdOx1 nCoV-19 vaccine was safe and immunogenic. However, it was not known whether this vaccine or any other COVID-19 vaccine candidates were efficacious in protecting against COVID-19 disease. The first press release announcing the efficacy of any COVID-19 vaccine was Pfizer-BioNTech on 9 November 2020.^34^ Therefore, vaccine efficacy and supply were hypothetical at the time of the interviews, although AstraZeneca had announced a vaccine would be available on a not-for-profit basis.

The interviews were recorded, transcribed verbatim and analysed using NVivo Version 12. A codebook was developed iteratively by the team, and regular meetings were used to check for consistency in the use of codes and to gain agreement for new or changed codes. We conducted an intercoder reliability (ICR) test and were able to demonstrate agreement between three coders was excellent on average (0.75+kappa value). The sample size we used for the ICR test was 70% of the total transcripts, as best practice taken from Campbell *et al*,^35^ and Lacy and Riffe,^36^ and we used NVivo to run a coding comparison query. We then used a constructivist grounded theory approach^37^ for our interpretation of the empirical material, following the principles of an iterative and reflexive process to identify themes as they emerged.^38^ This paper uses a selection of interview material relevant to the topic of analysis, and sections of transcripts are used as illustrative examples.

### Patient and public involvement

We consulted the Oxford Vaccine Group’s Public and Patient Involvement group for feedback on the study protocol and participant-facing materials before submitting the project for ethical approval. We incorporated suggestions to improve the clarity of the survey and interview guide, as well as addressing sensitivities and best practice about demographic classifications, including income and employment. Results are disseminated to study participants via the Oxford Vaccine Group website and newsletter. No patients involved in the recruitment to and conduct of the study.

## Results

Our survey generated 349 responses and we conducted semistructured interviews with 102 participants (the full survey questions and interview guide are provided in online supplemental appendix 1). The survey respondents were quite evenly split by sex—female (55%, 191/349), and 45–55 years was the biggest age group (33%, 114/349). Most respondents reported their nationality as British (84%, 292/349), 8% (28/349), reported being European and 2% (8/349), as American. Therefore, our sample was in line with the non-British born population in the UK, which was estimated in 2019 to make up 14% of the population.^39^ Other nationalities included New Zealand, Mexican, Filipino, Canadian and Japanese. Most respondents identified as white British (77% 267/349), with only 6% (21/349) described their ethnicity as Black, Asian and Minority Ethnic (BAME). These figures show that ethnic minority representation in the sample was low, as the national figure according to 2011 UK Census data is 13% that belong to a BAME group.^40^ Therefore, the lack of representation is a study limitation and is rooted in historical disparities in clinical trial participation by ethnic minority populations. Other studies have noted evidence of mistrust by ethnic minorities as a barrier to the willingness to participate in clinical trials and difficulties for representative recruitment^41 42^ as well as issues with the acceptability of vaccines.^43 44^ We also want to note the discrepancies in COVID-19 vaccine access and uptake, highlighted early in the pandemic through surveys assessing willingness to vaccinate, and the need for targeted vaccine-acceptance messaging.^45 46^ These factors mean that the ethnic minority views about vaccine nationalism and how this might relate to certain ethnic groups may not be adequately covered in this research.

10.1136/bmjgh-2021-006305.supp1 Supplementary data

**Figure 1:**
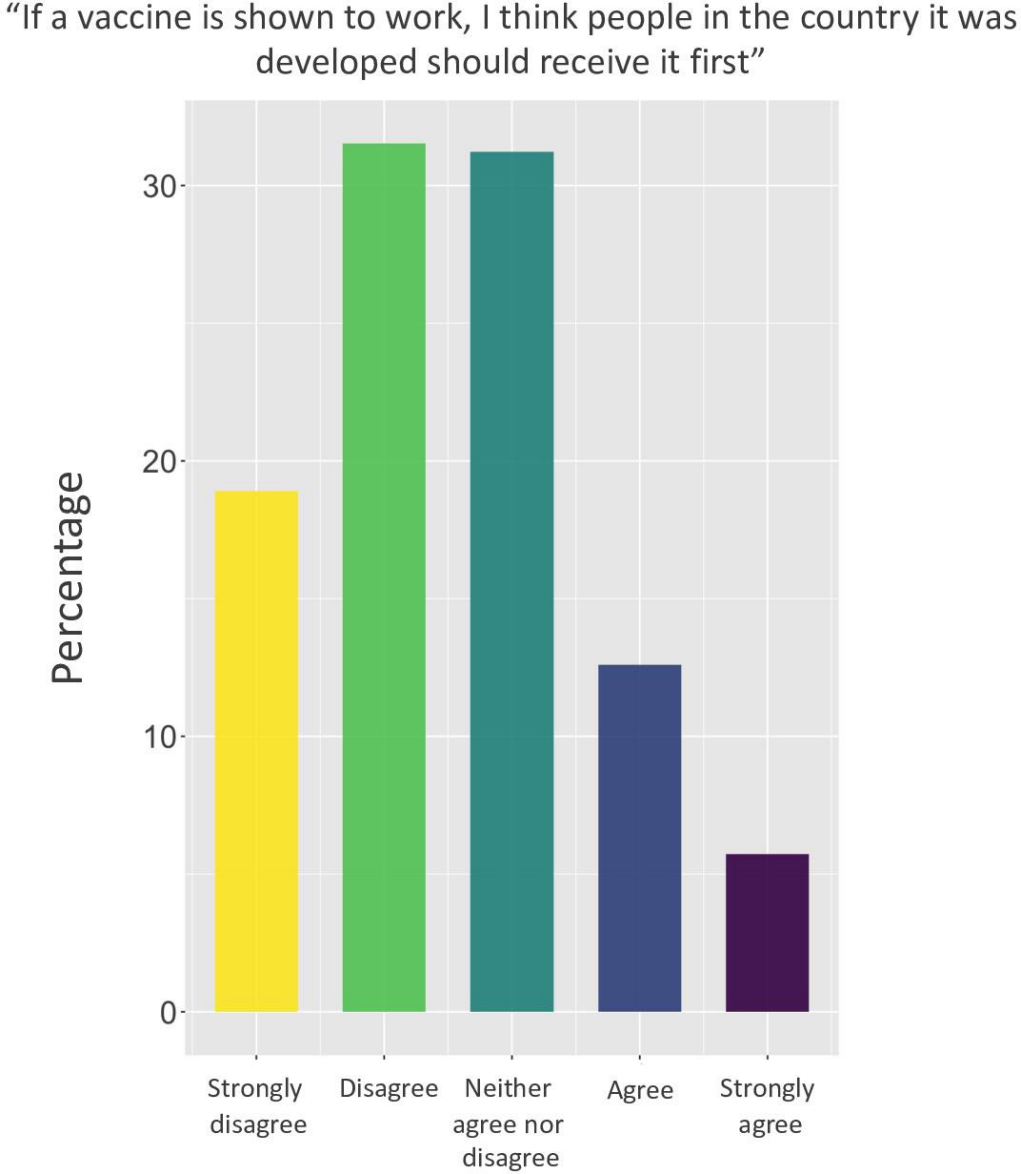
Survey question on vaccine priority.

More than half of respondents (56%, 194/349) were educated to postgraduate level and were employed full time 56% (197/349), with education, law and government services most commonly reported occupational groups. 40% (138/349) were living with a partner, 50% (173/349) were single and 62% (216/349) were without children. In the survey, respondents were asked to indicate their agreement to the statement: ‘If a vaccine is shown to work, I think people in the country in which it was developed should receive it first’. See the answers in figure 1.

Some suggested that the effectiveness of the vaccine would influence their thoughts: ‘*Global v National first is a hard one to decide without knowing how effective it is individually or by a large proportion of population to stop the spread’* (CQS-65615811) and: ‘*it also depends on its type of effectiveness - is it working best as preventing you personally from getting Covid or is it more working to prevent spread as that could affect who you prioritise’* (CQS-66013736). However, eight respondents commented in free text to express how they hoped for equitable global access. See a selection of responses below:

Preserving human life should be the priority of this vaccine and it should be administered to the most vulnerable globally as opposed to national/western dominance in the interest of economic recovery (CQS-65477606).

I think it should be given to those who will benefit the most from it, regardless of where in the world they are - any vaccine developed is a result of cumulative scientific research over decades and contributed to by people all over the globe (CQS-65501460)

As this is a global pandemic it should be given to those globally who are most at risk from the disease and keyworkers. As I see it until all countries take responsibility in the same way we will be living with COVID for a long time (CQS-65512171)

Others also explicitly noted their worries about commercial property and profit arrangements for the vaccine. They expressed their desire for the vaccine as ‘*not be protected commercial property’* (CQS-65502554), ‘*non-profit’* (CQS-65514001), ‘*not used just to make a profit’* (CQS-65599423), *‘minimum profit/cost per dose’* (CQS-65525489), ‘*distributed at cost’* (CQS-65497562) or that ‘*richer countries should probably pay more’* (CQS-65565429) to aid global access. In contrast, there were those who saw practical barriers to international ideals:

In ideal world a working vaccine should be made available globally ASAP, but somewhere has to be first and there might well be practical reasons as well as selfish ones for the country that develops it to be first to give it widespread. And it also depends on other vaccines and how they are progressing (CQS-66013736).

The idea of citizenship was invoked, although in different ways. One respondent was of the view that a vaccine should be shared globally but only after British citizens were vaccinated—echoing the responsibility argument of Ferguson and Caplan to (tax-paying) citizens:

I think as the taxpayer has contributed to the development of the vaccine, British citizens should be vaccinated first, although I obviously agree with sharing the vaccine globally in the long run (CQS-65486270).

Another respondent who was not originally from the UK saw taking part in the trial as part of a duty in becoming a British citizen:

I became a British citizen in 2020 (dual nationality Briitsh [sic] /American) and felt it was a civic duty (CQS-65681772).

In addition, two participants did not like the idea of the US having control of a vaccine:

I would be angry if Trump bought the rights to get it first, espcially [sic] as US is a first world country and has access to care” (CQS-65500690) and “Don't [sic] let Trump benefit from it politically (CQS-65499481).

### National and international themes

Through semi-structured interviews, participants were asked an open-ended question: Should UK citizens receive a vaccine first if it was the country to develop it? The individuals interviewed provided varied responses, but there were a number of themes that dominated: (1) national pride, (2) a global outlook, (3) prioritisation of vaccine supplies, (4) pessimism for a global solution, (5) politicisation.

### National pride

Interestingly, participants engaged in discussions on vaccine nationalism, before a vaccine was approved for emergency use, and before vaccine nationalism became an extensively covered topic in the media. It was clear that vaccine trial participants attached a national characterisation to the development of the Oxford-AstraZeneca vaccine. As Wilson has noted: ‘In the UK, the great attention paid to the Oxford vaccine trial… seems to partly stem from national pride’.^47^ The vaccine became a national metaphor by virtue of being generated through what was seen to be British science and scientists.

An element of national pride was expressed by the participants, and this was conceptualised in terms of ‘being better,’ ‘out-competing others’ or ‘winning’ as part of a race or competition. Although there was also a recognition of this being the general representation of the UK public even if the participants did not necessarily agree:

I think there is a thing about the UK being at the forefront that people really love… it shouldn’t be a competition but actually it is, for a lot of people it is (CQI-0343).

[The Oxford Vaccine Trial] It’s one that is being used to display the success or otherwise of the British response to the pandemic (CQI-0426).

It would be really, really exciting to be a part of something that has been created in the country where you live in (CQI-0471).

Participants suggested that the vaccine would lead the UK to be perceived more favourably by the rest of the world: ‘*that’s going to shine a spotlight on the UK’* (CQI-0471). One saw the vaccine as a triumph for humanity rather than the UK, ‘*if somewhere else got it first then great. This is a kind of humanity thing’* (CQI-0394). Another emphasised the importance to them that vaccine development took place locally. ‘*I live within the area. And I don’t sound like I grew up from Oxford, but I still think it would be amazing if Oxford got there. From the word go I’ve rooted for it’* (CQI-0394).

### A global outlook

More participants commented on having an international or global outlook on the pandemic, suggesting that long-term protection from COVID-19 would require populations in all countries to be vaccinated.

Participants commented on ethical, economic, practical and human costs of the pandemic worldwide, describing COVID-19 as a ‘*massive global destroyer of lives and economies’* (CQI-0471). Participants thought vaccines should be shared because the pandemic is a global problem:

I think we definitely need to try and have a global picture of this pandemic as a whole. The alternative would be to shut our borders and vaccinate everyone and then wait for the rest of the world to do their thing. But that’s probably not the right answer in terms of world economics, and probably ethics as well (CQI-0386).

It’s a worldwide problem, so I’d be happy if we have a worldwide solution, I don’t know how the distribution is going to be done. I guess I would feel a bit bleak if we were at the bottom of the list, but I don’t have any problem with sharing it with other nations (CQI-0304).

I just, from an ethical point of view, but also from a practical point of view, I just think it would be wrong to have a sort of UK-first policy (CQI-0312).

That is the essence of vaccination programmes. It’s a global problem and the vaccine should be a global resource (CQI-0379).

Through self-reflection, some participants, however, came to the conclusions that such a response, while optimal, may be idealistic and unrealistic:

You leave out the African countries or other poor countries, it’s just going to be a problem that bites us on the bum further down the line… I know it’s going to be a race; I know the reality of these things; it’s going to be each country for themselves probably. And America tries to buy out the stocks of everything at a particular moment. Yes, there’s a reality of the thing, but the idealist bit of me would just want a global approach to this really, because that’s the only way we’re going to solve it properly (CQI-0479).

Participants described a ‘fair’ global rollout where countries were able to access vaccines together:

I find it hard to justify how you would say one country should get it over another (CQI-0360).

I think the fairest way to distribute it is all at the same time. So, the UK, if there are 100 million ready to go, then let’s have like 30 million here and 70 million distributed elsewhere or whatever. It seems fair to me (CQI-0359).

### Prioritisation of vaccine supplies

In terms of how rollout is prioritised within populations, participants spoke of the need to target ‘*the most vulnerable across the world’* (CQI-0388). A number of participants thought the vaccine should go to where the need was greatest, which might be LICs with weaker healthcare infrastructure; or to individuals with the highest risk of infection, including key workers and the elderly.

These opinions were given before the UK Joint Committee on Vaccination and Immunisation (JCVI) published their advice on priority groups for COVID-19 vaccination on 30 December 2020^48^ :

It should go where and when the sort of need is (CQI-0370).

I think it should just go to globally whoever’s going to benefit from it the most… I think it should be key workers around the world, the people that need it the most around the world (CQI-0367).

I would be looking at where abroad people don’t have healthcare, and therefore you also ought to be saying, we don’t have enough ventilators for people, we don’t even have basic healthcare in these countries, that’s actually where the vaccine ought to be going (CQI-0435).

It should go to the countries with the greatest need first. Perhaps countries with ageing populations or with populations where the risk of infections still remains incredibly high (CQI-0363).

Two participants discussed the need for collaboration, both for equitable vaccine access and technology transfer, so that other countries could have the ability to produce vaccines for themselves (as proposed by COVAX):

If the UK is successful to develop it, that it [the UK] should be working very quickly alongside other countries and they can pick up the capabilities themselves and roll it out globally as fast as possible. I think the only way to do that is to be genuinely collaborative and open with our findings and share them (CQI-0455).

I don't really think that just because it was developed here, we should have first dibs… Yes, that’s a really difficult one ethically because there’s a lot of countries. The UK is really a very wealthy country so we can afford to mitigate the circumstances of the pandemic for most of the population. Whereas there are lot of other countries that, A. can't afford to produce their own vaccine, and are way behind if they tried now, which have far more fragile economies, far more people living in underprivileged circumstances. I struggle to say that the UK should be the first to get it, but also, I wouldn't know where to actually send it first. I think there was an initiative started recently where countries will give places like, I think, African countries, etc., doses. There’s a whole initiative about, I think, providing doses for countries like that. I definitely support that (CQI-0349).

Others suggested that the country developing a vaccine should have priority due to the use of taxpayer or government money, and the country facilities, talent and research:

I suppose if the trial’s funding has come from the UK government, then maybe that would be fair because it’s our taxpayers’ money that went into it (CQI-0306).

I know it’s a bit quite controversial but yes I think this should be a preference for the country that helped develop it. Because if that doesn’t happen then in the future nobody will want to spend the money paying for research, and the facilities, and paying for the research to be carried out. I think there should be a sight advantage for the country that carried it out, slight time advantage or money advantage, low cost but yes, I think there should be (CQI-0311).

I think it seems only fair that it has been UK resources and UK talent that has developed something that would work. I think it would be nice. But there has to be enough to then roll out to other countries and have it because if you don’t vaccinate the whole world, there’s potential for it to come back. But I would like to think that if it has been the UK one that actually works that you do have a little bit of privilege and say us first (CQI-0481).

Some participants admitted that they would like the UK to receive the vaccine first, so that life could return to normal life but as one participant noted, it did not mean to them that the lives of others were less important:

There is [sic] my own sort of thoughts about well, as it impacts my life, the more quickly people get vaccinated in the UK, the more quickly we can go back to life as normal, which is what I want (CQI-0403).

I mean, obviously, on the selfish side of things, I would like to be able to go outdoors again. But I mean, I don't believe that our lives are any more important (CQI-0472).

### Pessimism for a global solution

The acknowledgement of a discrepancy between an ideal solution and what occurs in reality is a theme that has historically been a feature of global goals, presented as an aspirational ideal when a high level of cooperation and coordination is required for countries to override national self-interests. Idealism is a long-held concept in international relations, towards ideas, goals and practices that are considered to be impractical in their realisation because of a ‘pessimistic reading of human nature along with an historical judgment on the difficulty of peaceably achieving radical change in world affairs’^49^ (Peter Wilson in Dowding 2011).

Participants reflected these ideas too, in the *‘hope that there would be a global solution’* (CQI-0306) as *‘something for humanity*’ (CQI-0438) but that it would not *‘happen logistically’* (CQI-0306) and *‘that’s idealistic and unlikely to play out that way’* (CQI-0438). Arguments for why a global solution would not be possible were proposed as being because ‘*governments invest in things they're going to want a certain percentage of for their population’* (CQI-0472).

One participant saw practical logistical barriers in developing countries being able to distribute vaccines and politically the need for the UK to be first:

Clearly, it should go to the places which need it most at the time, which actually UK is probably quite high up on the list. But certainly, when you're talking about the developing world, we have to work out how you get it distributed there as well …I think politically, it would have to be the UK. Although, it would have to be simultaneously ramped up everywhere. And that might not be fair. I think that’s just political reality (CQI-0433).

### Politicisation

The Independent Panel for Pandemic Preparedness and Response (IPPPR) has reported on the failings of global governance for COVID-19^50^. As Wenham describes, ‘Almost every section of the report points to the extent to which politics has driven the trajectory of the pandemic in different locations’.^51^ Wenham also warns that this politicisation of responses means that effort towards standardised global responses is a major challenge because the agreement of all countries is needed.

Similarly, many of the trial participants remarked on the politicisation of vaccines by politicians who used the vaccine as a nationalist metaphor for British greatness. For example, some regretted that if a vaccine was successful, the British politicians, including the Prime Minister, would use the opportunity to bolster the national image:

I can almost imagine if this vaccine is successful, I can honestly imagine Boris Johnson talking about bold Britain and blah, blah, blah, and that’s not something I want to see. But I can imagine that exactly happening. It’ll try and turn it into a nationalistic example of bold Britain (CQI-0438).

If in a few months there’s a press conference of Boris Johnson going around and be like, we, the British people, went around and did this amazing thing for the world, I think I would be just a bit pissed off (CQI-0404).

Others reflected on the political pressure for the Trump administration to produce a vaccine before the 2020 presidential elections and how strategically it would be a difficult position if other countries produced vaccines first:

America seems to be wanting to get one out before the election… And then on top of that, there’s the strategic considerations of the Americans want one, the Chinese want one. Europe can’t be in a position where it’s only America and China who have rushed out a vaccine and they’re having to buy or be gifted a vaccine from America in exchange for quid pro quo political horse-trading (CQI-0493).

The national metaphor of the Oxford-AstraZeneca vaccine as a ‘UK’ vaccine was questioned by one respondent who saw the large contribution of non-UK nationals and migrants to the trial and the vaccine development:

In fairness I find it funny, this idea that the UK developed a vaccine. I mean it’s true the vaccine is being developed in the UK, but most people I’ve interacted with in the vaccine trials have been foreigners and migrants. Obviously, the British government is funding some of it, there’s some British people involved, but this idea that the British population should have got given the right to more vaccines because it just happened to be done here seems a bit far-fetched to me (CQI-0404).

While participants viewed a vaccine developed by the UK as being trustworthy, they exhibited mistrust towards vaccines developed by other countries—especially where they were sceptical of the political regime, the rapid development of vaccines or the reliability of the data produced. Participants noted that they would be warier of a vaccine developed by Russia, China or the US, whose interests were seen to be politically driven and also an example of vaccine nationalism for these countries.

Another participant trusted the UK rather than the US to produce a vaccine at a fair price: *I prefer it to be developed in the UK, rather than the US, so it isn't massively exploited and sold to countries for millions of pounds* (CQI-0375). Of note at this point of time, the activities of vaccine diplomacy from Russia and China were not yet evident:

Probably, also, the one that’s in Russia. I’m not really sure what the laboratory is, but people are not going to trust that one, and the ones in China, as well (CQI-0308).

Russia’s decided it’s already got one. God knows how many corners they cut to do that. And China knows that if they can do it, they’re ahead, and will have a step ahead in front of America. And all of that going on. So, I don’t really know much about them, the ones that are in the lead, except they seem to be in the lead for other spurious reasons (CQI-0493).

There are two countries on trials that I would not take part on. Not if you paid me. And I think that’s all I need to say on that. They’ve been highly publicised, and there are two. And there’s one that, in The Lancet, that the data does look a little, a little, I’m trying to think of the word. Not quite right. So, one of them was published in The Lancet… But yes, the data just looks a little too linear (CQI-0394).

If it comes from China, maybe people won’t want it. I don't know (CQI-0436).

## Discussion

The uniqueness of this research has meant that the ability to make comparisons to other literature is limited and our discussion relies on drawing out the insights from the vaccine trial participants. Participants in this study suggested that national pride does not have to be at odds with a global outlook. The dichotomy between ‘national’ and ‘international’ in the context of vaccines and their supply are misconstrued as binary choices and perspectives. Many viewed a global outlook as being ethically favourable and also idealistic and not necessarily obtainable, and so harboured a pessimism for a global solution. The precise mechanisms of a global solution (such as COVAX) were not discussed in detail by participants. However, participants did discuss the need for collaboration, for equitable vaccine access and technology transfer, so that other countries could have the ability to produce vaccines for themselves. Other expert commentators have gone further to make proposals to counter vaccine nationalism, for example, through a ‘global compact’ to develop adult vaccination programmes, uninterrupted manufacturing capability and production capacity by private pharmaceutical companies.^52^

Participants were aware of the politicisation of vaccines and how that meant national objectives could be pursued over global equitable access. A small number of participants did echo the argument that UK citizens should receive a vaccine first. The national pride of participants has also been repeated in media reporting, although that has often been separately expressed to the issue of vaccine nationalism, which tended to only concern the supply of vaccines. Also, the pride that the participants spoke of related mostly to being part of something they saw as benefiting the greater good and creating a solution to the pandemic. Metaphorical representations and portrayals of national character are aspects of vaccines that the trial participants describe well and provide insight into—how science, scientific achievement and a vaccine developed and tested in the UK is seen through a national lens. This discussion goes beyond the current academic depictions of vaccine nationalism and has been achieved through the reflection of a public group closely involved in the development of COVID-19 vaccines during the pandemic.

Limitations of this paper come from interviewing a small subsection of the population and having a low number of ethnic minority participants in the study. Unfortunately, poor demographic representation has been an acknowledged issue in the recruitment of participants for clinical trials, as shown by several studies (notably by Flores *et al*).^53^ These participants in an early phase COVID-19 vaccine trials are personally invested in the vaccine development process and may have opinions that do not represent the wider public. Also, UK participants’ views may not be the same as participants in COVID-19 vaccine trials in other countries, particularly given likely differences in socioeconomic backgrounds. Furthermore, this paper is also highly influenced by the political context at the time and the relatively early phase of the pandemic when the study took place. To summarise, our key findings are shown below.

### National pride

Vaccine trial participants attached a national characterisation to the development of the Oxford-AstraZeneca vaccine and an element of national pride was expressed. There was also a recognition of this being the general representation of the UK public even if all the participants did not necessarily agree. Participants suggested that the vaccine would lead to the UK being perceived more favourably by the rest of the world, as a triumph for humanity rather than only the UK.

### A global outlook

Participants commented on having an international or global outlook to the pandemic, suggesting that long-term protection from COVID-19 would require populations in all countries to be vaccinated as part of the ethical, economic, practical and human costs of the pandemic worldwide. Participants thought vaccines should be shared because the pandemic is a global problem. Through self-reflection, some participants came to the conclusion that such a response was idealistic and unrealistic, despite thinking that equal access to vaccines constituted a ‘fair’ global rollout.

### Prioritisation of vaccine supplies

In terms of how rollout is prioritised within populations, participants spoke of the need to target the most vulnerable and where the need was greatest in the world. Participants discussed the need for collaboration, both for equitable vaccine access and technology transfer, so that other countries could have the ability to produce vaccines for themselves (as proposed by COVAX). Others suggested that the country developing a vaccine should have priority due to the use of taxpayer’s money and country research. Or they admitted that they would like the UK to receive the vaccine first, so that life could return to normal life but, it did not mean to them that the lives of others were less important.

### Pessimism for a global solution

Participants reflected ideals for a global solution but thought that it would not be practical and was idealistic. Arguments for why a global solution was not possible were proposed as being because of the political need for the UK to be first or practical logistical barriers in developing countries being able to distribute vaccines.

### Politicisation

Many of the trial participants remarked on the politicisation of vaccines by politicians who used the vaccine as a nationalist metaphor for British greatness, including the national metaphor of the Oxford-AstraZeneca vaccine as a ‘UK’ vaccine and trusting the UK rather than the US to produce a vaccine at a fair price. Others reflected on the political pressure for the Trump administration to produce a vaccine before the 2020 presidential elections and how strategically it would be a difficult position if other countries produced vaccines first.

## Conclusion

We have engaged with the current debate about vaccine nationalism but aimed to present the views of those who have contributed to the development of vaccines as participants in a COVID-19 vaccine trial. We, therefore, offer a unique perspective on questions about how vaccines should be prioritised, who should benefit, how these issues are discussed, as well as national and international approaches to the supply of vaccines. Of note, participants raised issues that subsequently became significant topics in media, academic and policy circles. This observation leads us to consider trial participants as an informed public group given their proximity to and knowledge of a vaccine trial. This group has been able to reflect deeply on the related social and political issues surrounding vaccines, which could be argued to represent a situated public expertise. The global community could draw on this expert opinion base to contribute to a focused public debate, rather than only opting for traditional routes of public and expert input, which are by nature often nation-state centred. The results of this research, therefore, will be of interest beyond those who follow the issue of vaccine nationalism and the views of vaccine trial participants.

What are the future implications of vaccine nationalism? The type of vaccine nationalism that is described in this paper is strongly attached to a vaccine developed in the UK having a national character, rather than only national pride. When a vaccine becomes a symbol to represent a nation, and those metaphors of scientific achievement and winning an international race are deeply engrained, it becomes more difficult for ownership of a vaccine to be international, based on need across the world. Longer term consequences of vaccine nationalism could mean that individual countries ensure that they invest in and expand national vaccine development and manufacturing capabilities. Such a move could be welcome on the one hand, given vaccines have arguably been underinvested in compared with other pharmaceutical products. On the other hand, an approach that is nationally focused could risk a widening of inequalities for those countries that cannot expand their own national capabilities. Where these developments might leave LMICs and the platforms that have been devised to attempt to provide equitable access are yet to be seen. COVAX is the initiative that encompasses the desire for a cooperative mechanism to distribute vaccines internationally based on need. However, the outcomes in practice are still falling far short of the vision. If a more international perspective is to be taken in the years to come in response to this continuing pandemic and for future pandemics, responsibility and the fulfilment of commitment are required for participating countries to share the benefits from collective action fairly. Equitable access to COVID-19 vaccines is not just a moral issue but is essential for the health security and economies of the global community. Public support emphasising the obligation for countries to share scientific benefits will remain important during and after the COVID-19 pandemic.

## Data Availability

No data are available.
