# Supplementary material for: Vaccine nationalism and internationalism: perspectives of COVID-19 vaccine trial participants in the United Kingdom
Source: BMJ Glob Health. 2021 Oct 18;6(10):e006305. doi: 10.1136/bmjgh-2021-006305 (PMC8526520; doi:10.1136/bmjgh-2021-006305)
Supplement: Supplementary data [file bmjgh-2021-006305supp001.pdf]

Online Supplemental Appendix 1

Understanding the Motivations and Experiences of Individuals Volunteering to Participate in a COVID-19 Vaccine Trial

SURVEY PARTICIPANT INFORMATION SHEET AND SURVEY

This project has been reviewed by, and received ethics clearance through, the University of Oxford Central University Research Ethics Committee R70147/RE001.

Why are we doing this survey?

The Oxford Vaccine Group is investigating public attitudes towards participation in COVID-19 vaccine trials. You are invited to participate in a separate research study about the motivations and views of individuals who volunteer to take part in COVID-19 vaccine trials. Where relevant, we are also interested in understanding the experiences of those participants who went on to participate in the vaccine trial. We hope the study findings will help us better understand trial participation and inform future trials and engagement about vaccines. If you are currently participating in 'A Study of a Candidate COVID-19 Vaccine' (COV001: REC reference: 20/SC/0145. IRAS project ID: 281259) your participation in this survey, and the responses you give to survey questions, won't affect your involvement or your upcoming clinic visits.

This survey will ask some questions that you have already answered during the screening process for the COV001 vaccine trial (e.g. your gender and ethnic group). This is because your answers to this current questionnaire are completely separate and unlinked to your data included in the vaccine trial.

Do I have to take part?

Taking part is voluntary. You can withdraw at any point for any reason, up to the submission of your answers by closing the browser. Please note that once you have finished completing the survey, we will be unable to identify and remove your data, as all the information you provide is recorded without direct identifiers. You will not receive any remuneration for taking part and your participation in this interview, and the responses you give won't affect your involvement in the COV001 Vaccine Trial.

During this survey

You will be asked to read and answer questions about your motivations to take part in the UK COVID-19 vaccine trial. It should take about 15-20 minutes. No background knowledge is required. We would like to follow up this survey with interviews (up to 1 hour each) via video call or phone. If you would be willing to take part in an interview, at the end of the survey we

provide further information and if you are happy to take part we will ask you for an email address so that we can contact you (not everyone will be contacted for an interview).

How will my data be used?

The research team (including the researchers and transcribers) will have access to the research data. Responsible members of the University of Oxford may be given access to data for monitoring and/or audit of the research.

- Your answers to the survey will not include direct identifiers and we will take steps to make sure that your responses remain confidential. However, if you take part in an interview after the survey, your responses to the survey will have the potential to be linked to your contact details.
- Your data will be stored on password-protected and encrypted research computers.
- Your data will only be used for non-commercial purposes, to inform academic research and publications.
- Your IP address will not be stored.
- Research data will be stored for three years after publication or public release of the findings of the research. Because the data will not include direct identifiers we will not be able to act upon any individual responses to the survey.
- The University of Oxford is the data controller with respect to your personal data (contact details, if you provide them), and as such will determine how your personal data is used in the study. The University will process your personal data for the purpose of the research outlined above. Research is a task that we perform in the public interest.
- Further information about your rights with respect to your personal data is available from https://compliance.admin.ox.ac.uk/individual-rights.
- Your personal information and data collected in this study will not be shared with other researchers or organisations.

What if there is a problem?

If you have a concern about any aspect of this project, please contact us on covid-19-qual@ox.ac.uk and we will do our best to answer your query. We will acknowledge your concern within 10 working days and give you an indication of how it will be dealt with. If you remain unhappy or wish to make a formal complaint, please contact the Chair of the Medical Sciences Interdivisional Research Ethics Committee at the University of Oxford: ethics@medsci.ox.ac.uk (Research Services, University of Oxford, Wellington Square, Oxford OX1 2JD).

How do I find out about the results?

We will provide information about the results of this study through the Oxford Vaccine Group website (https://www.ovg.ox.ac.uk/).

COVID-19 Qualitative Study Team

Many thanks in advance for your time.

COVID-19 Qualitative Study Team

Please note that you may only participate in this survey if you are 18 years of age or over.

☐ I certify that I am 18 years of age or over.

If you have read the information above and agree to participate with the understanding that the data (including any personal data) you submit will be processed accordingly, please check the relevant box below to get started.

☐ Yes, I agree to take part

I. DEMOGRAPHICS

1) What is your gender?
Male
Female
Other
Prefer not to say

2) What is your age group?
Under 25
25-34
35-44
45-55

3) Nationality
Drop down list.

4) What is your ethnic origin?
White - British
White - Irish
White - Other
Black or Black British - Caribbean
Black or Black British - African

Black or Black British - Other
Asian or Asian British - Indian
Asian or Asian British - Pakistani
Asian or Asian British - Bangladeshi
Asian or Asian British - Chinese
Asian or Asian British - Other Asian background
Mixed - White and Black Caribbean
Mixed - White and Black African
Mixed - White and Asian
Mixed - Other
Gypsy or Traveller
Arab
Other
Prefer not to say

5) What is the highest level of education you have completed?
GCSE
A-Level or equivalent
Bachelors or equivalent
Postgraduate
Prefer not to say
Other…

6) Current Employment status
Employed full time (40 or more hours per week)
Employed part time (up to 39 hours per week)
Unemployed and currently looking for work
Unemployed and not currently looking for work
Student
Retired
Homemaker
Self-employed
Unable to work
Furloughed

7) What is your usual occupation?
Management
Business/Finance
Natural/applied science
Health
Education, law, social, government services
Sales and services
Trades and transport
Student

Unemployed
Retired
Other

8) In which of these groups is your yearly household income?
Less than £15,000
£15,000 - £24,999
£25,000 - £54,999
£55,000 - £99,999
£100,000 - £149,999
£150,000 or more
Prefer not to say

9) Have you been financially disadvantaged by the COVID-19 pandemic?
Yes
No
I don't know

10) At the time of the trial screening, where were you living?
Living with relatives/ family
Living alone
Living with partner
Living in a university room
Living in a shared house
Temporarily staying with friends
Other ...

11) Marital status
Single (never married)
Married, or in a domestic partnership
Widowed
Divorced
Separated
Prefer not to say

12) How many children do you have?
None
1
2-4
More than 4
Prefer not to say

13) Have you participated in a clinical trial before?
Yes

No
Prefer not to say

14) How did you hear about the vaccine trial? Mark all that are relevant.
Friend
University email
Internet search
University/OVG/Jenner website
Radio
TV
News article
Social media
Other …

- If social media, which form?
  Facebook
  Twitter
  Reddit
  Instagram
  LinkedIn
  Whatsapp
  Other

II. ATTITUDES & BELIEFS

Views on COVID-19, research and the pandemic

- I think COVID-19 poses a serious threat to health.
    - Strongly agree
    - Agree
    - Neither agree nor disagree
    - Disagree
    - Strongly disagree

- I think COVID-19 poses a serious threat to the economy.
    - Strongly agree
    - Agree
    - Neither agree nor disagree
    - Disagree
    - Strongly disagree

- I think a vaccine is the main solution to the COVID-19 pandemic.
    - Strongly agree
    - Agree
    - Neither agree nor disagree
    - Disagree

- Strongly disagree

- I think participation in COVID-19 vaccine trials should be mandatory.
  - Strongly agree
  - Agree
  - Neither agree nor disagree
  - Disagree
  - Strongly disagree

- Clinical trial research is essential to improve the prevention and control of disease.
  - Strongly agree
  - Agree
  - Neither agree nor disagree
  - Disagree
  - Strongly disagree

- Clinical research is the basis for most improvements in healthcare.
  - Strongly agree
  - Agree
  - Neither agree nor disagree
  - Disagree
  - Strongly disagree

<u>Before this study, what was your knowledge of clinical trials? Tick as many as apply to you.</u>

- ○ I had never heard of them
- ○ I knew a little bit about them
- ○ I knew quite a bit about them
- ○ I knew a lot about them
- ○ I work, or have worked, in clinical trials
- ○ I work, or have worked, in research

<u>Do you have any other comments about COVID-19 or research in a pandemic?</u>
...

III. <u>MOTIVATIONS I</u>

<u>What were your motivations to take part in the trial?</u>

- I wanted the chance to get the vaccine as it might give me protection against COVID-19.
  - Strongly agree
  - Agree
  - Neither agree nor disagree
  - Disagree

- Strongly disagree

\- I was joining a friend or family member who was participating.

- Strongly agree
- Agree
- Neither agree nor disagree
- Disagree
- Strongly disagree

\- I felt a responsibility to take part.

- Strongly agree
- Agree
- Neither agree nor disagree
- Disagree
- Strongly disagree

\- I wanted to access health care/medical attention.

- Strongly agree
- Agree
- Neither agree nor disagree
- Disagree
- Strongly disagree

\- I wanted to be part of something important.

- Strongly agree
- Agree
- Neither agree nor disagree
- Disagree
- Strongly disagree

\- I wanted to have a new experience.

- Strongly agree
- Agree
- Neither agree nor disagree
- Disagree
- Strongly disagree

\- I was curious about the trial.

- Strongly agree
- Agree
- Neither agree nor disagree
- Disagree
- Strongly disagree

Vanderslott S, *et al. BMJ Global Health* 2021; 6:e006305. doi: 10.1136/bmjgh-2021-006305

- I wanted to receive attention.

- Strongly agree
- Agree
- Neither agree nor disagree
- Disagree
- Strongly disagree

- I wanted to report my experience to the media.

- Strongly agree
- Agree
- Neither agree nor disagree
- Disagree
- Strongly disagree

- I wanted to receive financial compensation.

- Strongly agree
- Agree
- Neither agree nor disagree
- Disagree
- Strongly disagree

- I wanted to tell others about my experience.

- Strongly agree
- Agree
- Neither agree nor disagree
- Disagree
- Strongly disagree

- I thought that if I caught COVID-19 infection and needed to go to hospital I would have preferential treatment.

- Strongly agree
- Agree
- Neither agree nor disagree
- Disagree
- Strongly disagree

IV. <u>MOTIVATIONS II</u>

<u>What were your motivations to take part in the trial?</u>

- I wanted to contribute to the improvement of the health of others.

- Strongly agree
- Agree
- Neither agree nor disagree
- Disagree
- Strongly disagree

Vanderslott S, *et al. BMJ Global Health* 2021; 6:e006305. doi: 10.1136/bmjgh-2021-006305

- I wanted to contribute to scientific progress.
  - Strongly agree
  - Agree
  - Neither agree nor disagree
  - Disagree
  - Strongly disagree

- I felt that others would view my participation positively.
  - Strongly agree
  - Agree
  - Neither agree nor disagree
  - Disagree
  - Strongly disagree

- I wanted to advance the development of the vaccine.
  - Strongly agree
  - Agree
  - Neither agree nor disagree
  - Disagree
  - Strongly disagree

- I wanted to help my community/society/country/world.
  - Strongly agree
  - Agree
  - Neither agree nor disagree
  - Disagree
  - Strongly disagree

- I thought it would have a positive effect on my wellbeing.
  - Strongly agree
  - Agree
  - Neither agree nor disagree
  - Disagree
  - Strongly disagree

- I wanted a reason to leave the house.
  - Strongly agree
  - Agree
  - Neither agree nor disagree
  - Disagree
  - Strongly disagree

- I wanted to take action/make a difference/achieve something.

- Strongly agree
- Agree
- Neither agree nor disagree
- Disagree
- Strongly disagree

\- I felt powerless during the COVID-19 pandemic.

- Strongly agree
- Agree
- Neither agree nor disagree
- Disagree
- Strongly disagree

<u>Did you have any other motivations/reasons for taking part in the trial?</u>

...

<u>Incentives</u>

\- The financial compensation was my main motivation for taking part in this trial.

- Strongly agree
- Agree
- Neither agree nor disagree
- Disagree
- Strongly disagree

\- I would have taken part in this trial without any compensation.

- Strongly agree
- Agree
- Neither agree nor disagree
- Disagree
- Strongly disagree

\- The amount of financial compensation for participation in the trial was appropriate.

- Strongly agree
- Agree
- Neither agree nor disagree
- Disagree
- Strongly disagree

\- I would have preferred to receive compensation in the form of shopping vouchers.

- Strongly agree
- Agree
- Neither agree nor disagree
- Disagree
- Strongly disagree

Do you have any comments about financial incentives in the trial process?

...

V. MOTIVATIONS III

I would have participated in the trial if it also involved…

- Being infected with COVID-19 and having to isolate from others.
  - Strongly agree
  - Agree
  - Neither agree nor disagree
  - Disagree
  - Strongly disagree

- Being exposing to someone who was infected with COVID-19.
  - Strongly agree
  - Agree
  - Neither agree nor disagree
  - Disagree
  - Strongly disagree

- Being infected with COVID-19 but only with significant financial compensation.
  - Strongly agree
  - Agree
  - Neither agree nor disagree
  - Disagree
  - Strongly disagree

Do you have any further comments about the questions above?

…

VI. RISKS

I was worried about the risk of taking part in a vaccine trial during the pandemic because:

- Vaccines developed during outbreaks are riskier.
  - Strongly agree
  - Agree
  - Neither agree nor disagree
  - Disagree
  - Strongly disagree

- New vaccines are riskier than ones that have been around for a while.

Vanderslott S, *et al. BMJ Global Health* 2021; 6:e006305. doi: 10.1136/bmjgh-2021-006305

- Strongly agree
- Agree
- Neither agree nor disagree
- Disagree
- Strongly disagree

- My personal privacy could be compromised.
  - Strongly agree
  - Agree
  - Neither agree nor disagree
  - Disagree
  - Strongly disagree

- My security or safety could be compromised.
  - Strongly agree
  - Agree
  - Neither agree nor disagree
  - Disagree
  - Strongly disagree

<u>I think this trial is riskier because:</u>

- It is the first time this vaccine has been given to humans.
  - Strongly agree
  - Agree
  - Neither agree nor disagree
  - Disagree
  - Strongly disagree

- Some of the tests usually required for a newly manufactured vaccine have been modified.
  - Strongly agree
  - Agree
  - Neither agree nor disagree
  - Disagree
  - Strongly disagree

- There is a quick turnaround from results of vaccination in animals to the first injection in humans.
  - Strongly agree
  - Agree
  - Neither agree nor disagree
  - Disagree
  - Strongly disagree

- A large number of people are being vaccinated over a short space of time.
  - Strongly agree
  - Agree
  - Neither agree nor disagree
  - Disagree
  - Strongly disagree

<u>I have been worried that:</u>

- I could catch COVID-19 when attending the study clinic for vaccination or follow-up visits.
  - Strongly agree
  - Agree
  - Neither agree nor disagree
  - Disagree
  - Strongly disagree

- I could have a severe reaction to the COVID-19 vaccine.
  - Strongly agree
  - Agree
  - Neither agree nor disagree
  - Disagree
  - Strongly disagree

- I could have a severe reaction to the Men ACWY (control) vaccine.
  - Strongly agree
  - Agree
  - Neither agree nor disagree
  - Disagree
  - Strongly disagree

- I could catch COVID-19 as a result of attending clinic visits
  - Strongly agree
  - Agree
  - Neither agree nor disagree
  - Disagree
  - Strongly disagree

- I could catch COVID-19 and have worse symptoms than those individuals who have not been vaccinated.
  - Strongly agree
  - Agree
  - Neither agree nor disagree
  - Disagree
  - Strongly disagree

- There could be long term health implications from the vaccine.
  - Strongly agree
  - Agree
  - Neither agree nor disagree
  - Disagree
  - Strongly disagree

- There could be financial impacts from participating in the trial e.g. if I got sick and could not work.
  - Strongly agree
  - Agree
  - Neither agree nor disagree
  - Disagree
  - Strongly disagree

- I could be subject to high media interest due to trial involvement.
  - Strongly agree
  - Agree
  - Neither agree nor disagree
  - Disagree
  - Strongly disagree

- Participating in the trial may affect my health insurance.
  - Strongly agree
  - Agree
  - Neither agree nor disagree
  - Disagree
  - Strongly disagree

<u>Are there any other risks you are/were worried about?</u>
…

<u>Do you have any further comments about the risks of the trial?</u>
...

VII. <u>OUTCOMES</u>

- I am confident that this vaccine will work.
  - Strongly agree
  - Agree
  - Neither agree nor disagree
  - Disagree

- Strongly disagree

- If the vaccine is shown to work, I think health workers, key workers and 'at risk' groups should receive the vaccine first.
  - Strongly agree
  - Agree
  - Neither agree nor disagree
  - Disagree
  - Strongly disagree

- If the vaccine is shown to work, I think it should be shared globally, with those at the highest risk receiving it first.
  - Strongly agree
  - Agree
  - Neither agree nor disagree
  - Disagree
  - Strongly disagree

- If a vaccine is shown to work, I think people in the country in which it was developed should receive it first.
  - Strongly agree
  - Agree
  - Neither agree nor disagree
  - Disagree
  - Strongly disagree

<u>Do you have any further comments about the outcomes of the trial?</u>
...

<u>ENROLMENT QUESTION</u>

<u>Were you vaccinated during the study?</u>
Yes/No (if yes, routed to VIII; if no, routed to IX)

VIII. EXPERIENCES IF ENROLLED

1) <u>Which study group are you in?</u>

2) <u>Which trial centre did you attend?</u>
   (drop down list of cities)

3) <u>Which vaccine do you think you have received?</u>
   COVID-19 vaccine
   Men ACWY vaccine (the control)

I don't know

4) How have you found being part of the trial?
   - It has been an entirely positive experience
   - It has been a mostly positive experience
   - It has been a neutral experience
   - It has been a mostly negative experience
   - It has been an entirely negative experience

5) Please give further details on your answer to Question 4.
   …

6) Could anything be improved about how the trial process has been conducted so far?
   …

IX. EXPERIENCES IF NOT ENROLLED

1) Which trial centre did you attend?
   (drop down list of cities)

2) What reason was given for not enrolling you in this trial (i.e. not receiving a vaccination)?
   Medical history
   Results of blood/urine tests
   My observations (blood pressure, temperature, pulse) were too high or low
   Alcohol/cigarette/drug consumption
   I live with a vulnerable person
   I had symptoms of COVID-19 or had self-isolated since February
   I could not get to the vaccine centre without using public transport
   I no longer wished to participate
   Prefer not to say
   Other ...

3) How did you find the screening process?
   - It was an entirely positive experience
   - It was a mostly positive experience
   - It was a neutral experience
   - It was a mostly negative experience
   - It was an entirely negative experience

4) Please give further details on your answer to Question 3.
   ...

5) <u>How did you feel about not participating further in the trial? Tick all that apply.</u>
   I didn't mind
   Relieved
   Disappointed
   Sad
   Angry
   Other …
   - Why did you feel this way? (as a sub question) …

6) <u>Could anything be improved about how the trial process was conducted?</u>
   ...

X. FURTHER PARTICIPATION

Would you be interested in receiving more information about participating in an interview for a more detailed discussion of your experiences on the trial (this would be conducted remotely and not face to face)?
Y v N

If Y
This participant information sheet provides the information about the interview section of this study and how your data will be used. If you are happy to be contacted please provide your contact details below. If you have any questions please contact the study team at covid-19-qual@ovg.ox.ac.uk

Name:
Email Address:
Phone number:

COVQUAL Survey v 1.0 26th Aug 2020 and COVQUAL Structured Interview Guide v 1.0 26th Aug 2020

INTERVIEW GUIDE

Oral Information Giving for Semi-Structured Interviews

This project has been reviewed by, and received ethics clearance through, the University of Oxford Central University Research Ethics Committee R70147/RE001.
Ensure the environment in which the meeting takes place is conducive to maintaining the privacy of the participant during the interview.
Ensure that verbal consent is taken.
Record using a digital recorder (if the participant has consented to this).

Interview
Introduction:
Hello [x], my name is [x] from the University of Oxford, Oxford Vaccine Group COVID-19 Qualitative Study team.

Let me explain what will happen during this interview, once we start the recording I will read you a short paragraph about this study. Then I will ask you to confirm that you have read the information sheet and are happy to take part. I will also introduce the interview with you using your interview participant number.

I will just highlight a few key points about the interview.
- I will be recording the audio from our interview onto a digital recorder and the recording will be stored securely using your study number.
- If you would like to end the study at any point do let me know.

Are you happy for me to start recording? [Await confirmation]

<START RECORDING>
This is an interview with participant number: CQIXXX on DD/MMM/YYYY

Many thanks for agreeing to speak with me today. The Oxford Vaccine Group is investigating public attitudes towards participation in vaccine trials. We hope this separate study will help better understand trial participation and inform future trials and engagement about vaccines. Participation in this qualitative study is voluntary and the responses you give won’t affect your involvement in the COV001 Vaccine Trial.

To allow us to continue can I confirm that you have read the interview participant information sheet. [Await confirmation].

Questions/concerns
Ask if they understand this before continuing and if they have any questions.

Can I confirm you consent to take part? [Await confirmation]. Ok, thank you.

Please could you tell me how old you are and how would you describe your gender?

(Please read the questions well beforehand and use them as a guide to structure the conversation. There is no need to read the questions word-for-word).

1. Views and experiences of COVID-19
- How has your experience of the COVID-19 pandemic been? - What have been the positive or negative aspects of the pandemic?
- When did you realise COVID-19 was a serious health issue?
- Have you been personally affected by COVID-19? Do you know anyone who has been ill with COVID-19?

2. Motivations
- What did you see as the advantages of volunteering for the COVID-19 vaccine trial?
- What are the disadvantages of volunteering for this trial?
- What improvements could be made about how the trial is performed?

3. Friends and family
- Did you ask anyone's opinion before taking part in the trial? Is there anyone else that you know taking part in the trial? Who did you tell about your involvement in the trial?
- Did your friends and family approve or disapprove of you taking part in the trial? Did you have any unanticipated responses from those you told of your involvement?
- What were their concerns or objections (if any)? What were their approvals or encouragements (if any)?
- What do you think the public perception of the trial is? Have you been communicating about the trial to others? Have you shared your participation on social media? If yes, have you received approving or disapproving responses?

(These answers may be affected by the social media guidance, as they've been asked not to engage, so might be less willing to admit)

4. Vaccine outcomes (concentrate on this vaccine)
- Do you think this vaccine will work? What were your thoughts before and after the publication of the initial findings?
- Do you think you have received the COVID-19 vaccine?
- Since being involved in the vaccine trial, have you noticed any changes in your behaviour? (e.g. increased/decreased social distancing)
- If the vaccine is not effective will the trial still have been useful?
- Would you agree to be intentionally infected after receiving a COVID-19 vaccine to test if it works (this is called a human challenge or controlled human infection model study)?
- If the vaccine is shown to be safe and effective vaccine, how do you think it should be deployed? If a vaccine is shown to work, do you think health workers, key workers, and 'at risk' groups should receive the vaccine first?
- If this vaccine is shown to work, do you think people in the UK should receive a vaccine first? What if it was made in the UK?
- Are you aware of other vaccine trials? If yes please provide examples.

5. Trial experience during pandemic
- What has your experience been taking part in this trial during lockdown? Have there been any particular constraints or benefits? What would be different if there was not a lockdown?
- Do you think trials should be conducted faster during outbreaks?
- Which parts of a trial do you think can be speeded up (animal testing, human trial, regulatory approval, ethics review, manufacturing)?
- Do you think a vaccine should be manufactured even if there is a chance it does not work?

6. Risk/Safety
- When you decided to take part in the trial, how risky did you think it was? What is an acceptable level of risk for taking part in a vaccine trial?
- Is animal testing necessary to develop vaccines and treatments?
- If you experience any adverse events what will you expect will happen?
- Do you think some people should not take part in this trial? For what reasons?
- What risk(s) did you think about/worried you most before you took part in the trial?
- Have you been worried that you could become seriously ill as a result of your participation in the study?
- Were you surprised that if you developed Covid-19 disease during the trial you would not be entitled to additional healthcare (ie beyond the NHS)?
- Do you think vaccines developed during outbreaks are riskier? Do you think new vaccines are riskier than ones that have been around for a while?

7. Financial incentive
- Was the financial compensation a motivation for taking part in this trial?
- Do you think the financial compensation should be: none, lower, the same, higher. Do you think the financial compensation is fair? What amounts would be too little or too high?
- Would you have taken part in this trial without any compensation?

8. Attitudes to vaccines
- Have you been fully vaccinated? And if you have children, have they been fully vaccinated? Are you supportive of all vaccination? Why do you think some people oppose vaccination?
- Do you know people who have been opposed to the vaccine or who you think would be?
- Do you agree with mandatory vaccination? Should a COVID-19 vaccine be made mandatory?

9. Before this study, what was your knowledge of clinical trials? (heard, or knew about them)
Have you been in a clinical trial before?
If yes - How many and what kind of trial?
Was that a positive experience?

10. Would you take part in other clinical trials?
- Yes – why?
- No – why?
- Don't know – [prompt: for example, a similar vaccine trial, a trial for other medicines, or this was a one-off, open to possibility]
- What would encourage you to take part in another trial? What would discourage you from taking part in another?

11. Media representation
- How do you think this trial has been represented in the media? Have you seen other trial participants in the media? What did you think?
- Have you been approached by the media during this trial? Have you engaged with the media? Do you intend to engage with the media? What has your experience of the media been?
- Have you been communicating about the trial on social media?
- Were you aware of any 'fake news' or misinformation surrounding the trial?

(May need to reassure that all answers are confidential and there is no right/wrong answer)

12. Trial progress/feedback
- Could anything be improved about how the trial is performed so far?
- What has been the best aspect of being in the trial? What has been the worse/most cumbersome aspect of being in the trial?

13. Conclusion
Is there anything else that you would like to comment on that I haven’t already asked you about? Or any questions about this study?

Thank you very much for your time and the information you shared today.

<STOP RECORDING>
